# A Novel Multidisciplinary Intervention for Long-Term Weight Loss and Glycaemic Control in Obese Patients with Diabetes

**DOI:** 10.1155/2015/729567

**Published:** 2015-04-08

**Authors:** Anna Lih, Lorraine Pereira, Ramy H. Bishay, Johnson Zang, Abdullah Omari, Evan Atlantis, Nic Kormas

**Affiliations:** ^1^Department of Endocrinology & Metabolism, Concord Repatriation General Hospital, Rhodes, NSW 2139, Australia; ^2^Sydney Medical School, University of Sydney, Sydney, NSW 2006, Australia; ^3^University of New South Wales Medical Program, University of New South Wales, Sydney, NSW 2052, Australia; ^4^Department of Vascular Medicine, St. Vincent's Hospital, Darlinghurst, Sydney, NSW 2010, Australia; ^5^School of Nursing and Midwifery, University of Western Sydney, Campbelltown, NSW 2560, Australia; ^6^School of Medicine, University of Adelaide, Adelaide, SA 5005, Australia

## Abstract

*Introduction*. Obesity and diabetes are difficult to treat in public clinics. We sought to determine the effectiveness of the Metabolic Rehabilitation Program (MRP) in achieving long-term weight loss and improving glycaemic control versus “best practice” diabetes clinic (DC) in obese patients using a retrospective cohort study. *Methods*. Patients with diabetes and BMI > 30 kg/m^2^ who attended the MRP, which consisted of supervised exercise and intense allied health integration, or the DC were selected. Primary outcomes were improvements in weight and glycaemia with secondary outcomes of improvements in blood pressure and lipid profile at 12 and 30 months. *Results*. Baseline characteristics of both cohorts (40 MRP and 40 DC patients) were similar at baseline other than age (63 in MRP versus 68 years in DC, *P* = 0.002). At 12 months, MRP patients lost 7.65 ± 1.74 kg versus 1.76 ± 2.60 kg in the DC group (*P* < 0.0001) and 9.70 ± 2.13 kg versus 0.98 ± 2.65 kg at 30 months (*P* < 0.0001). Similarly, MRP patients had significant absolute reductions in %HbA1c at 30 months versus the DC group (−0.86 ± 0.31% versus 0.12% ± 0.33%, *P* < 0.038), with nonsignificant improvements in lipids and blood pressure in MRP patients. *Conclusion*. Further research is needed to establish the MRP as an effective strategy for achieving sustained weight loss and improving glycaemic control in obese patients with type 2 diabetes.

## 1. Introduction

Obesity is a serious condition strongly associated with excess dietary intake and physical inactivity. It is estimated that 205 million men and 297 million women older than 20 years worldwide were obese in 2008 [1]. The prevalence of obesity in Australia almost tripled since 1980 [1] and results from the Australian Health Survey estimate that 28.3% of adults were obese in 2011 [2]. One of the consequences of rising prevalence of obesity globally has been the concomitant increase in type 2 diabetes mellitus (T2DM) [3]. Indeed, the International Diabetes Federation (IDF) estimated that 366 million people worldwide, or 8% of adults, now have diabetes [4].

Once comorbid, diabetes significantly increases the health and economic burden of obesity, being the most common reason for renal dialysis, blindness in people less than 60 years of age, nontraumatic lower-limb amputation, and cardiovascular disease [4]. The rising health burden of T2DM disproportionately affects older, obese, and physically inactive people [5]. Conversely, weight loss has been demonstrated to significantly improve glycaemic control in obese patients with diabetes, and this relationship is most evident in bariatric studies with remission of diabetes achieved in 73% of surgical patients versus 13% in a conventional therapy group [6]; this outcome was likely due to a number of factors in addition to weight loss such as altered delivery of nutrients, gut hormone changes, and alterations to bile acid and amino acid metabolism [7]. Nonetheless, lifestyle changes aimed at achieving a healthy body weight and moderate physical activity should be the initial intervention for all obese patients with pre- and established diabetes [8–10].

Weight loss is complex in obese patients with diabetes where many antidiabetic medications can increase weight, especially insulin [11]. Additionally, sustained weight loss is difficult to achieve as most obese patients regain the majority of the weight initially lost within 2–5 years [12, 13]. The problem of clinically meaningful and sustainable outcomes was also gleaned from the recent cessation of the Look AHEAD trial [14], which, despite achieving good 1-year weight losses of 8.6% of initial body weight in the intervention group, only very modest losses were achieved at 10 years (6%).

Current “best practice” diabetes clinics (DC) utilizing medical, nursing, and limited allied health support are the current standard of care for managing patients with T2DM. However, it is questionable whether the DC offers the best care long-term in obese patients, especially since obesity is associated with reduced responsiveness to blood glucose lowering medications over time, and in turn increased medication and insulin dosages may induce further weight gain, perpetuating the problem [16, 17].

With some exceptions [18–20] there is paucity of data evaluating long-term outcomes in obese patients in diabetes clinics. We have recently reported that weight loss and improved glycaemic control can be achieved and maintained over a 3-year period in a small population of patients enrolled in an intensive, multidisciplinary lifestyle intervention called the Metabolic Rehabilitation Program (MRP), the first of its kind in Australia [21]. However, the long-term benefits of the MRP compared with “best practice” DC are unknown. We hypothesized that obese patients with T2DM enrolled in the MRP would achieve significant weight loss and improved glycaemic control when compared with DC patients and that these results would be durable long-term using a retrospective cohort study design.

## 2. Materials and Methods

### 2.1. Study Design

**Figure 1:**
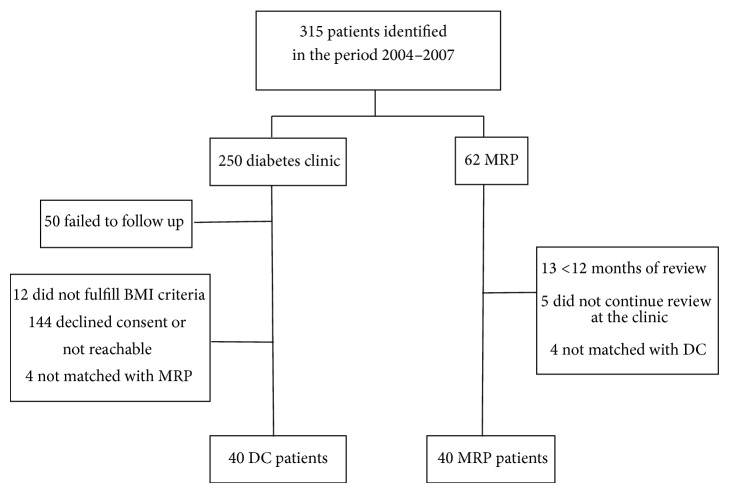
Study participants selection and case-matching.

This was a retrospective cohort study conducted at Concord Repatriation General Hospital, a major teaching hospital and tertiary referral center in Sydney, Australia. In all, 40 MRP patients met inclusion criteria and were case-matched with 40 DC patients with respect to BMI, age, gender, glycaemic control, and duration of diabetes (Figure 1). Exclusion of participants included those who could not be contacted to obtain consent, BMI less than 30 kg/m^2^, and/or inability to verify anthropometric and medical history. Diagnosis of T2DM was established if patients had fasting plasma glucose equal to or greater than 126 mg/dL (7.0 mmol/L) or a 75 g OGTT consistent with a diagnosis of diabetes. Participants were enrolled in either the MRP or the DC for a minimum of 12 months between the periods from 2004 to 2007 and had follow-up clinical data for 30 months. The study was approved by the Sydney South West Area Health Service Human Research Ethics Committee; all patients were contacted to obtain informed consent for study inclusion.

### 2.2. Metabolic Rehabilitation Program (MRP)

The MRP was established in 2003 and designed specifically as a comprehensive lifestyle program or “one-stop shop” for managing complex obese patients with T2DM. All patients in the MRP had a diagnosis of T2DM and BMI greater than 30 kg/m^2^ and were at least 18 years of age. Exclusions included type 1 diabetes mellitus, pregnancy, previous bariatric surgery during the study period, eating disorders, major comorbidity limiting physical exercise, or conditions associated with unintentional weight loss such as malignancy, HIV, dialysis, or home oxygen.

The primary intervention of the MRP was twofold: to achieve significant and sustainable weight loss and improved glycaemic control. This program, outlined previously [21], which included a collaborative multidisciplinary team supervised by an endocrinologist (NK) specializing in obesity and diabetes was conducted monthly for 6 months and then every 2-3 months thereafter. Allied health integration consisted of review by a dietician upon enrolment into the MRP, with follow-up consultations on a 6-weekly basis and an annual review. Concurrently, there were also eight group sessions conducted to assist patients with implementing individualized diet programs and meal replacements (e.g.,* Optifast*). In addition, there was a clinical review by a diabetes educator upon enrolment and subsequently twice a year. An on-site psychologist was available for an initial consultation and four group sessions conducted throughout the program. The exercise program was supervised by physiotherapists and exercise physiologists for 6 days a week to prescribe and supervise exercise. Weight losing medications were not used. Oral hypoglycaemic pharmacotherapies were titrated at the discretion of the treating endocrinologist based on national guidelines current at the time of the study [22]. The educational component of the MRP was adopted from the Bodylines program, a validated educational weight loss program used successfully in previous studies [23].

The intensive exercise program consisted of 330 minutes of moderate intensity physical activity a week. This comprised a compulsory 180 minutes of supervised exercise classes at an on-site gymnasium in the hospital and a further 150 minutes on off-clinic days. Exercise intensity was based on achievement of a heart rate (HR) response of 60–80% of predicted maximal HR (average range 110–140 beats per minute), taking into consideration age, fitness level, risk of injury, and cardiorespiratory risk factors. The prescribed exercises included 20–30 minutes each of aerobic exercises (treadmills, biking, step-up boxes, and walking) and resistance training (pin-loaded machines and free weights). Whenever possible, the supervising endocrinologist would reduce weight-promoting antiglycaemic medications and substitute them with weight neutral medications as clinically indicated or cease medications altogether.

### 2.3. Diabetes Clinic (DC)

Diabetes clinic patients were seen at the outpatient hospital clinic and managed by optimizing glycaemic control and risk factor modification of hypertension and dyslipidaemia and mitigating long-term micro- and macrovascular complications of diabetes using pharmacological and nonpharmacological methods based on “best practice” guidelines at the time of the study [22]. The DC consisted of consultations with endocrinologists (AL) specializing in diabetes and obesity medicine at 3–6 monthly intervals and regular but less frequent appointments with the same diabetes nurse educators and dietitians as in the MRP. Referrals to other outpatient services and specialists such as psychologists, podiatrists, ophthalmologists, nephrologists, and cardiologists were carried out as required based on clinical guidelines at the time of study.

### 2.4. Patient and Laboratory Assessment

The interval of medical reviews was consistent between the two groups, with patients being reviewed at the start of clinical encounter and then at 3–6 monthly intervals. The collection of data for all primary and secondary outcomes was obtained at baseline, 12 months, and 30 months. Our primary outcome data included weight (kg), BMI, and %HbA1c whilst our secondary aims included total, high-density (HDL) and low-density (LDL) lipoprotein cholesterol, total triglycerides, and blood pressure. The calculation of BMI was determined by the standard formula weight (kg) divided by height (meters) squared. Improvement in glycaemic control was defined as greater than 1% reduction. The commercial assay used was the HPLC assay (BIORAD Variant II TURBO). An improvement in weight was defined as a significant decrease whereas successful weight loss was defined as 5% or more of baseline weight lost, both at either 12 or 30 months [24]. Percentage change in all anthropometric and laboratory data was calculated as the measured value minus baseline value divided by baseline value and multiplied by 100%.

### 2.5. Statistical Methods

This study was retrospective and sought to recruit 90 participants in total. It was determined that this number of participants would provide the study with a power of 80% to detect a clinically important reduction of ≥5% in weight, with a two-sided type 1 error of 5% and a potential withdrawal rate of 10%. Descriptive analysis was performed on the baseline demographic and anthropometric characteristics between the selected patient groups. To determine differences between the MRP and DC groups, *χ* ^2^ test was used for categorical variables and the Student's *t*-test and Mann-Whitney *U* test were used for continuous variables. Analysis of covariance (ANCOVA) was used to determine group differences adjusting for covariates of age, gender, and BMI. A *P* value < 0.05 was considered statistically significant. All statistical analyses were performed using SPSS Statistics Version 19 (IBM Corporation, Armonk, NY, USA).

## 3. Results

Between 2004 and 2007, 80 participants were included in the analysis (Figure 1). The baseline characteristics of the participants are described in Table 1. The mean age of participants in the DC group was 5 years more than those enrolled in the MRP (68.3 ± 9.2 versus 63.3 ± 8.5 years), *P* = 0.002. However, the majority of patients were between the ages of 51 and 80 years (45% in both groups). The BMI, gender distribution, and duration of diabetes were not significantly different between the groups. Initial glycaemic control was poorer in the MRP group than the DC group (%HbA1c of 8.2 ± 1.6 versus 7.9 ± 1.9 or 66.3 ± 17.5 versus 63.2 ± 20.8 mmol/mol, resp.) though this difference was nonsignificant.

At 12 months, the MRP group lost significantly more weight compared to the DC cohort (7.65 ± 1.74 kg versus 1.76 ± 2.60 kg, Table 2). At 30 months, MRP patients achieved a mean weight loss of 9.70 ± 2.13 kg versus lesser gains in the DC patients of 0.98 ± 2.65 kg (*P* < 0.0001). In the DC group, 13% of participants regained weight versus 8% in the MRP group.

Glycaemic control was markedly improved in the intervention group versus the DC group at 12 months, with absolute percentage-point reduction in %HbA1c of 0.95 ± 0.28 versus 0.35 ± 0.34, respectively (*P* = 0.08). The improvement in glycaemic control continued at 30 months, with a significant reduction in %HbA1c of 0.86 ± 0.31 whilst the DC group had a reduction of 0.12% ± 0.33 (*P* < 0.038).

Participants that lost weight had improved glycaemic control; 60% of participants that lost weight demonstrated improved %HbA1c at 12 months. Those patients that reached successful weight loss (≥5%) at 12 months were greater in the MRP versus the DC cohort (35% versus 10%, *P* < 0.001). This improvement was also more likely to be maintained long-term at 30 months in the MRP versus the DC group (36% versus 12%, *P* < 0.001).

At 30 months, participants who achieved a %HbA1c less than 5.7 were found exclusively in the MRP (representing 3.9% of all patients) and achieved >5% weight loss. For %HbA1c within a range of 5.7–6.5, 7.8% of participants achieved this target range in both the DC and MRP treatment arm and all had documented weight loss, albeit some fell short of 5% weight loss at 30 months.

There was also a reduction in systolic blood pressure (SBP) of 9.50 ± 3.28 mmHg in the MRP group versus 0.77 ± 3.91 mmHg in the DC cohort at 12 months, though this was not statistically significant in this small study. There was also a statistically significant improvement in HDL levels at 30 months as well as nonsignificant improvements in LDL, triglycerides, and diastolic blood pressure.

## 4. Discussion

This study demonstrated that an intensive multifaceted lifestyle intervention was superior for achieving long-term weight loss and improvements in glycaemic control in obese adults with type 2 diabetes compared with traditional “best practice” in a diabetes clinic, in the Australian public health system. Our findings should inform both diabetes and obesity practitioners and health policy decision-makers in Australia.

Remission of diabetes in obese patients is now thought to be possible and has been achieved in those who have achieved long-term weight loss following bariatric surgery, as demonstrated by the randomized control study by Dixon et al. and the more recent STAMPEDE study [6, 25, 26]. Similarly, using nonsurgical approaches, severe caloric restriction (600 kcal/day) in 11 obese patients with diabetes demonstrated reversal of beta cell dysfunction and remission of diabetes with a reduction of %HbA1c from 7.4 to 6 [27]. In our study employing nonsurgical, intensive integration of allied health, 60% of participants that lost weight demonstrated improved %HbA1c at 12 months, with MRP participants achieving more successful weight loss (≥5%) at 1 year versus the DC cohort, and were nearly three times more likely to be maintained long-term. Although not measured specifically in this study, our small study raises the possibility of diabetes remission in a population of obese patients using a nonsurgical approach.

Traditional diabetes clinics often lack a structured weight management program and there is little data evaluating the efficacy of traditional diabetes clinics for obese people with diabetes. One study [28] evaluated a diabetes clinic with a structured weight management program in a community hospital and achieved a %HbA1c of less than 7% in 42% of patients, while other studies emphasizing optimal blood pressure and lipid profile targets achieved a %HbA1c less than 7% in 20% of patients at 30 months [29, 30]. This highlights the importance of weight management in diabetes clinics. Our small study revealed that even obese patients with diabetes for an appreciable duration and mostly in their 6th decade of life can achieve an absolute reduction of nearly 1 point in %HbA1c at 1 year and 0.86 after 30 months or %HbA1c of 7.25 and 7.34, respectively, with structured exercise and integration of dietetics and physiotherapy.

Weight loss, in particular long-term losses, is difficult to achieve in obese patients with diabetes due to many factors. However, modest weight loss in diabetic patients at 12 months has been previously demonstrated [31, 32] whilst other studies have shown rebound weight gain of up to 50–66% within two years [11], while only few studies have shown sustained long-term weight loss. The Finnish study [18], a three-year trial which randomized obese patients (BMI 31.4 kg/m^2^) with impaired glucose tolerance to either conventional therapy or intensified lifestyle modification program, showed only modest weight loss of 3.5 kg (versus 9.7 kg in our study). A study by Brown et al. compared weight loss achieved with either dietary versus behavioural interventions in a 2-year trial and demonstrated that dietary interventions achieved significantly more weight loss (9 kg versus 3 kg, resp.) [33] than behavioural interventions, emphasizing the importance of a structured eating plan and the synergistic effect of combined behavioral and dietetic interventions as employed by our study. Our study achieved superior weight loss, with MRP patients losing on average 7.65 ± 1.74 kg at 12 months compared with 1.76 kg in the DC group. Furthermore, in comparison with previous trials, our patient cohort were able to maintain significant weight loss up to 30 months with 9.7 ± 2.13 kg in the MRP compared with only 0.98 ± 2.65 kg in the DC. This not only demonstrates the long-term effectiveness of the MRP but also suggests the need for successful programs to be continued beyond 2 years as rebound weight gain is often seen in this time period.

The Look AHEAD study [34, 35] is one of the largest trials evaluating intensified lifestyle programs directed at obese diabetes patients. The group aimed for a reduction in weight of ≥10% at 12 months in enrolled participants. The profile of patients enrolled in this intensified program had a mean BMI of 36 kg/m^2^ and HbA1c of 7.2%. The participants in our study in both groups had a mean BMI of 37 kg/m^2^ and mean HbA1c of 8%, reflecting the higher metabolic burden in our patients. The achieved weight loss at 3 years was 5.5% in the intensified treatment arm, in contrast to the MRP group, which achieved weight loss of 9.8% at 30 months. We have previously commented that this apparent discrepancy may be due to the greater amount of prescribed and supervised exercise sessions of participants in the MRP group [21] and is substantiated by the work of prior studies [36–38] which have advocated for the importance of exercise in maintaining glycaemic control in patients with diabetes.

Many studies have failed to show long-term amelioration of cardiometabolic risk factors to 30 months [12, 39]. Our study demonstrated reductions in systolic blood pressure, LDL cholesterol, and triglycerides as well as an increase in HDL cholesterol, although, with the exception of HDL, these did not reach statistical significance between groups. This was most likely due to multiple factors including pharmacological unloading, anticipated increase in blood pressure following initial weight loss [40], and the small study sample. Furthermore, our study did not evaluate adherence to lipid lowering and antihypertensive pharmacological agents and represents a reasonable avenue for a larger, more rigorous study.

Several limitations of our study warrant further consideration. The results of this retrospective study were based on two small cohorts reviewed over 30 months in routine clinical practice. However, this study was adequately powered to detect clinically meaningful weight loss and is the first to have assessed the success of weight loss and glycaemic control long-term in specialist clinics in Australia. Other study limitations and potential sources of bias include the self-selection of patients into each intervention (nonrandom treatment allocation) and the fact that MRP patients were younger and perhaps more motivated. Socioeconomic status was also not directly studied and selected patients who were mostly limited resided within a 10 km radius of the study site.

There are many logistical and financial challenges in developing multifaceted programs with the goal of achieving long-term weight loss as well as limitations to the availability of human resources with expertise in weight management. Future implementation research should focus on facilitating the feasibility and applicability of intensive lifestyle intervention for diabetes and obesity management in the public health system, with increasingly diminishing resources. It is plausible that the cost of such programs may be offset by reduction in the burden of disease, the cost of treating complications of diabetes and obesity, and pharmacological unloading. Therefore, future studies employing a randomised controlled trial comparing the efficacy and cost-effectiveness of patients enrolled in the MRP with standard care or surgery, with a larger population size, longer follow-up, and data on pharmacological agents, especially given the era of newer oral hypoglycaemic agents (e.g., DPP-4 inhibitors, GLP-1 agonists, and SGLT2 inhibitors) would be invaluable. These studies may potentially change current guideline-driven models of care.

## 5. Conclusions

The findings of this study show that intensive multidisciplinary specialist care was more effective in achieving long-term weight loss and improving glycaemic control in obese patients with type 2 diabetes compared with “best-practice” diabetes clinic care. Further research on the feasibility and applicability of this model of care in other settings and countries is required to inform health policy-makers and specialist practitioners of the best “obesity and diabetes care” long-term.

## Figures and Tables

**Table 1:** Baseline characteristics of patients enrolled in the diabetes clinic (DC) and Metabolic Rehabilitation Program (MRP).

	National guideline targets^∗^	MRP *n* = 40	DC *n* = 40	*P* value
Gender		Female 55%	Female 51%	0.85
		Male 45%	Male 49%	
Mean age (years)		63.3 ± 8.5	68.3 ± 9.2	0.002^∗∗^
Weight (kg)		106.2 ± 18.2	100.8 ± 17.9	0.78
BMI (kg/m^2^)	20–25	37.5 ± 5.3	37.7 ± 6.3	0.59
HbA1c				
(%)	≤7% (range 6.5–7.5)	8.2 ± 1.6	7.9 ± 1.9	0.56
(mmol/mol)	≤53 (range 48–58)	66.3 ± 17.5	63.2 ± 20.8	
Duration of diabetes	≤10 years	21 (27.3%)	21 (27.3%)	0.74
	10 years	19 (24.7%)	19 (24.7%)	
SBP (mmHg)	<130	136 ± 18.6	141 ± 16.1	0.12
DBP (mmHg)	<80	79 ± 9.2	77 ± 11.1	0.51
HDL (mM)	≥1	1.3 ± 0.8	1.2 ± 0.3	0.29
LDL (mM)	<2.5	2.6 ± 0.9	2.6 ± 0.7	0.90
Triglycerides (mM)	<2	2.1 ± 1.0	2.2 ± 1.0	0.66

^∗^National guidelines are based on [15]. Values are shown as means (±SD). Significance is set at *P* < 0.05 and highlighted by asterisk (∗∗).

HbA1c, serum glycosylated haemoglobin expressed as % and mmol/mol; SBP, systolic blood pressure; DBP, diastolic blood pressure; HDL, serum high-density lipoprotein cholesterol; LDL, serum low-density lipoprotein cholesterol.

**Table 2:** Percent changes in weight, BMI, %HbA1c, and other clinical outcomes of patients enrolled in the Metabolic Rehabilitation Program (MRP) versus the diabetes clinic (DC) at 12 and 30 months.

	MRP n = 40	DC n = 40	P value

Weight			
Baseline (kg)	106.2 ± 18.2	100.8 ± 17.9	0.78
Δ12 months	−7.65 ± 1.74	−1.76 ± 2.60	<0.0001^∗∗^
Δ30 months	−9.70 ± 2.13	−0.98 ± 2.65	<0.0001^∗∗^
BMI			
Baseline (kg/m^2^)	37.5 ± 5.3	37.7 ± 6.3	0.59
Δ12 months	−7.73 ± 1.83	−2.23 ± 1.74	<0.0001^∗∗^
Δ30 months	−10.9 ± 1.9	−1.59 ± 2.2	<0.0001^∗∗^
ΔHbA1c			
Baseline (%, mmol/mol)	8.2 ± 1.6 (66.3 ± 17.5)	7.9 ± 1.9	0.56
Δ12 months	−0.95 ± 0.28	−0.35 ± 0.34	0.08
Δ30 months	−0.86 ± 0.31	−0.12 ± 0.33	0.04^∗∗^
ΔSBP			
Baseline (mmHg)	136 ± 18.6	141 ± 16.1	0.12
Δ12 months	−9.50 ± 3.28	−0.77 ± 3.91	0.15
Δ30 months	−7.51 ± 3.47	−3.37 ± 4.23	0.21
ΔDBP			
Baseline (mmHg)	79 ± 9.2	77 ± 11.1	0.51
Δ12 months	−5.20 ± 1.72	−3.49 ± 2.29	0.39
Δ30 months	−4.39 ± 1.93	−4.76 ± 2.22	0.82
ΔHDL			
Baseline (mM)	1.3 ± 0.8	1.2 ± 0.3	0.29
Δ12 months	−0.03 ± 0.16	−0.09 ± 0.06	0.14
Δ30 months	−0.049 ± 0.15	−0.07 ± 0.07	0.04^∗∗^
ΔLDL			
Baseline (mM)	2.6 ± 0.9	2.6 ± 0.7	0.90
Δ12 months	0.12 ± 0.19	−0.23 ± 0.18	0.35
Δ30 months	−0.37 ± 0.18	−0.35 ± 0.16	0.62
ΔTriglycerides			
Baseline (mM)	2.1 ± 1.0	2.2 ± 1.0	0.66
Δ12 months	−0.26 ± 0.23	−0.29 ± 0.21	0.98
Δ30 months	−0.53 ± 0.22	−0.11 ± 0.23	0.13

Values are shown as net change from baseline ± standard deviation (SD). Significance is set at *P* < 0.05 and highlighted by asterisk (∗∗). Abbreviations are as described in Table 1. The covariates of age, gender, and BMI were taken into account in the analysis.
